# Identification of a Chondrocyte-Specific Enhancer in the *Hoxc8* Gene

**DOI:** 10.3390/jdb12010005

**Published:** 2024-01-24

**Authors:** Stephania A. Cormier, Claudia Kappen

**Affiliations:** 1 Department of Respiratory Immunology and Toxicology, Pennington Biomedical Research Center, Louisiana State University System, 6400 Perkins Road, Baton Rouge, LA 70808, USA; stephaniacormier@lsu.edu; 2 Department of Developmental Biology, Pennington Biomedical Research Center, Louisiana State University System, 6400 Perkins Road, Baton Rouge, LA 70808, USA

**Keywords:** reporter transgene, LacZ, enhancer, combinatorial regulation, cartilage, perichondria, proliferating chondrocytes, mouse embryo

## Abstract

*Hox* genes encode transcription factors whose roles in patterning animal body plans during embryonic development are well-documented. Multiple studies demonstrate that *Hox* genes continue to act in adult cells, in normal differentiation, in regenerative processes, and, with abnormal expression, in diverse types of cancers. However, surprisingly little is known about the regulatory mechanisms that govern *Hox* gene expression in specific cell types, as they differentiate during late embryonic development, and in the adult organism. The murine *Hoxc8* gene determines the identity of multiple skeletal elements in the lower thoracic and lumbar region and continues to play a role in the proliferation and differentiation of cells in cartilage as the skeleton matures. This study was undertaken to identify regulatory elements in the *Hoxc8* gene that control transcriptional activity, specifically in cartilage-producing chondrocytes. We report that an enhancer comprising two 416 and 224 bps long interacting DNA elements produces reporter gene activity when assayed on a heterologous transcriptional promoter in transgenic mice. This enhancer is distinct in spatial, temporal, and molecular regulation from previously identified regulatory sequences in the *Hoxc8* gene that control its expression in early development. The identification of a tissue-specific *Hox* gene regulatory element now allows mechanistic investigations into Hox transcription factor expression and function in differentiating cell types and adult tissues and to specifically target these cells during repair processes and regeneration.

## 1. Background

The homeobox gene *Hoxc8* is expressed in the developing mouse embryo in the neural tube and mesoderm, as well as their derivatives [1,2,3,4,5]. Disruption of the *Hoxc8* gene by gene targeting produced mutants with homeotic transformations in the thoracic and lumbar skeleton [6], demonstrating that Hoxc8 is required during the patterning phase of skeletal development. Roles for Hoxc8 have also been described in motorneuron innervation and survival [7] and in the adult hematopoietic system, as evidenced by the reduced propensity for erythrogenic and granulocyte–macrophage differentiation in *Hoxc8* knockout mutant mice [8]. We have previously shown that Hoxc8 exerts a tissue-specific function in the developing skeleton by regulating chondrocyte differentiation and maturation [9]. When the *Hoxc8* gene was overexpressed in transgenic mice under the control of its own upstream region, profound cartilage defects were observed that were incompatible with survival. The rib cartilage of these mice exhibited increased proliferation of immature chondrocytes at the expense of cell maturation [9]. Consistent with a cell-type-specific function in chondrocytes, a reduction in *Hoxc8* expression in cultured primary rib chondrocytes by an antisense–oligonucleotide approach was associated with decreased cell proliferation due to prolonged transit through and delayed exit from the M phase [10]. These approaches provided evidence that Hoxc8, in addition to its function in skeletal patterning, plays a role in chondrocyte proliferation.

However, the molecular mechanisms controlling *Hoxc8* expression in tissue- and cell-type-specific fashion have not been identified to date. We and others showed previously that the 5′ upstream region flanking the *Hoxc8* promoter is sufficient to direct chondrocyte-specific expression of a reporter transgene [9,11], consistent with the earlier finding that a *LacZ* gene inserted into the *Hoxc8* locus by homologous recombination is expressed in the developing skeleton at birth [6]. In this study, we sought to identify the particular regulatory element(s) in the *Hoxc8* upstream region that control chondrocyte-specific gene expression. Previously, Shashikant et al. [12] characterized a 335 basepair long regulatory element that is active as an enhancer in the developing nervous system in a pattern that reflects the “early” domain of *Hoxc8* expression in the embryo. However, the activity of this “early” enhancer is downregulated in mesodermal derivatives prior to skeleton formation [12]. Thus, it appears that any mesoderm-specific, and particularly cartilage-specific, enhancer activity is controlled by different areas in the 5 kb *Hoxc8* upstream region. We, therefore, set out to identify the cartilage-specific regulatory element(s) using the *LacZ* reporter gene approach in transgenic mice. Analyses of reporter gene activity were performed at day 15.5 of gestation when skeletogenesis was well underway and chondrogenic cells were differentiating.

## 2. Methods and Materials

### 2.1. In Situ Hybridization

In situ hybridization was performed as described in Utset et al. [3]. Sections were cut to 10 μm thickness from paraffin-embedded mouse embryos isolated at 12.5 through 18.5 days of gestation. Sections were hybridized with 35 S-labeled riboprobes for *Collagen II* [13] and *Hoxc8* [3]. The *Hoxc8* probe detects the 5′ region of the RNA, excluding the homeobox.

### 2.2. LacZ Transgene Constructs

The *Hoxc8* upstream region fragments were cloned into the pIE-*LacZ* vector, in which the *LacZ* gene is linked to the minimal promoter from the Herpes Simplex Virus ICP4 gene [14]. We have previously demonstrated that this reporter is inactive in transgenic mice in the absence of an enhancer element in the construct [15]. The subcloning of fragments was performed using the XbaI site as the acceptor for blunt-ended inserts and XbaI and PstI or XhoI for directional cloning of inserts.

Constructs A and C were generated using the HindIII site at position 2116, and construct B was created using the NcoI site at position 3849. For constructs D through H, specific 5′ and 3′ primer pairs were designed that carried XhoI and XbaI sites, respectively, allowing directional cloning of an amplified and appropriately digested PCR product. Construct F + H was generated from construct D by combining PCR-generated fragments, Construct I was created by first designing complementary primers for each strand that span the deletion, followed by independent PCR in the respective 3′ direction using the respective end primers. In a third step, both PCR products were mixed and amplified using only the end primers, which also incorporated XbaI and PstI sites for directional cloning into the pIE-LacZ vector. All constructs were verified by DNA sequencing.

### 2.3. Generation and Analysis of Transient Transgenic Embryos

Microinjections were performed as described before [16], and FVB mice were used as embryo donors throughout the project. Embryos were isolated at day 15.5 of gestation, and yolk sacs or placentas were used for genotyping using primers specific for the *LacZ* transgene [15]. All procedures for analysis of β-gal expression were performed exactly as published before [15,16,17], with the exception that embryos were eviscerated, and fixation prior to staining was performed in 1% paraformaldehyde overnight; staining itself was also allowed to proceed overnight.

Reporter gene activity and staining patterns were deemed consistent and cartilage-specific when a given transgene construct produced the same staining pattern in at least three *LacZ* transgene-positive samples of progeny, each from independent pregnancies.

### 2.4. Histology

After microscopic inspection, β-galactosidase-positive embryos were embedded in paraffin for serial sectioning in order to enable inspection of the staining patterns at cellular resolution. Cross-sections of 10 μm thickness were produced, deparaffinized, and scored under DIC microscopy for the presence of β-galactosidase staining in skeletal structures and any other tissues.

### 2.5. Sequence Analysis

The sequence upstream of the mouse *Hoxc8* gene has previously been published [18] and is listed in the UCSC genome browser at coordinates 102,893,993–102,898,962 on chromosome 15 (mm31). The human and rat *Hoxc8* sequences were retrieved by searching with the *Hox3.2–3.1* intergenic region [18] from Genbank accession #M35603. Conserved regions were visualized using VISTA (http://genome.lbl.gov/vista/index.shtml accessed on 1 January 2023). Putative transcription factor binding sites were retrieved from the Jaspar database of curated information (http://jaspar.genereg.net,accessed on 1 January 2023), as visualized through the UCSC genome browser (https://www.genome.ucsc.edu accessed on 1 January 2023) using genome assembly mm10. Only motifs that were predicted by both the 2018 and the 2020 versions of Jaspar were considered in our analyses.

## 3. Results

**Figure 1:**
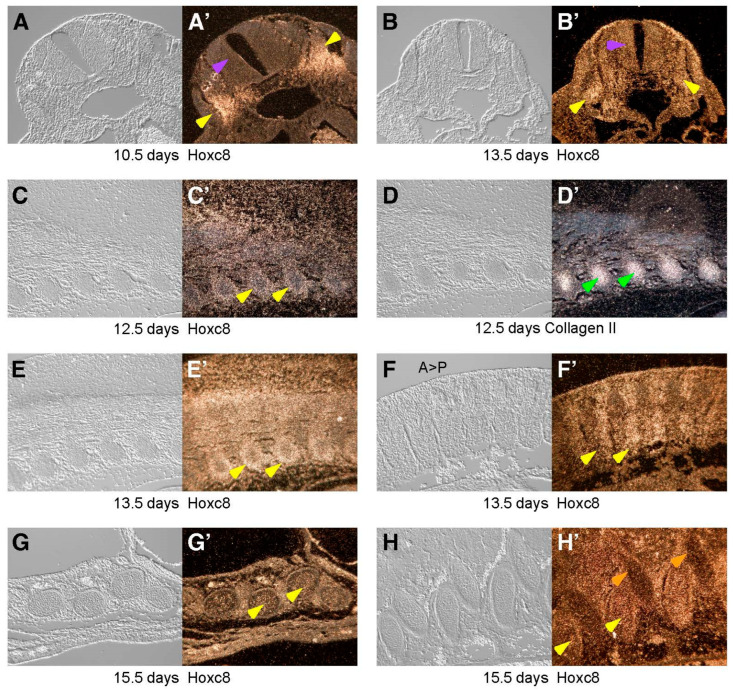
*Hoxc8* expression in the developing skeleton at 10.5–15.5 days post-coitum. *In situ* hybridization with probes specific for *Hoxc8* and *Collagen II* to sections of normal mouse embryos isolated at various developmental stages. The left panels (**A**–**H**) in each pair depict paraffin sections in brightfield under differential interference contrast, with the *in situ* hybridization on that section imaged under darkfield conditions and depicted in the corresponding right panels (**A’**–**H’**). (**A**,**A’**) At E10.5, strong *Hoxc8* expression is apparent in prevertebral cells (yellow arrowheads), while at this axial level, *Hoxc8* expression in the neural tube is low (purple arrowhead). (**B**,**B’**) Section through an embryo at E13.5 that displays *Hoxc8* expression both in the neural tube (purple arrowhead) and mesoderm (yellow arrowheads). (**C**,**C’**) At E12.5, *Hoxc8* expression is strongest at the periphery of condensing precartilaginous rib structures (yellow arrowheads) surrounding committed chondrocytes, as evidenced by *Collagen II* expression within the structures (green arrowheads) in the panels (**D**,**D’**,**E**,**E’**). At E13.5, *Hoxc8* expression becomes stronger, as prevertebral structures condense and undergo cartilage differentiation. (**F**,**F’**) Within each segment in the vertebral column on this parasagittal section, *Hoxc8* is more strongly expressed in the caudal part (yellow arrowheads, A>P indicates anterior–posterior direction). (**G**,**G’**,**H**,**H’**) At E15.5, *Hoxc8* expression is evident in ventral (**G**,**G’**) and dorsal (**H**,**H’**) portions of the ribs (yellow arrowheads) and in the periphery of the vertebral bodies (orange arrowheads).

The initial indication that the 5 kb fragment upstream from the *Hoxc8* promoter confers cartilage-specific enhancer activity came from experiments performed using the VP16-based binary transgenic system [9,15]. In this system, the *LacZ* transgene is activated by the viral transcriptional transactivator VP16. Driven by the *Hoxc8* 5 kb upstream fragment, β-galactosidase expression was observed specifically in the growth zones of the developing skeleton and cartilaginous structures. In order to further characterize the expression of the *Hoxc8* gene in developing cartilage, we performed in situ hybridization using a probe that is specific for the 5′ region of the *Hoxc8* mRNA. Figure 1 shows that during mouse development, *Hoxc8* expression is detected in the somitic mesoderm and later in skeletogenic cells that condense to form cartilage anlagen. By 15.5 days of gestation (E15.5), *Hoxc8* expression is detected in chondrocytes of the ribs and prevertebrae.

**Figure 2:**
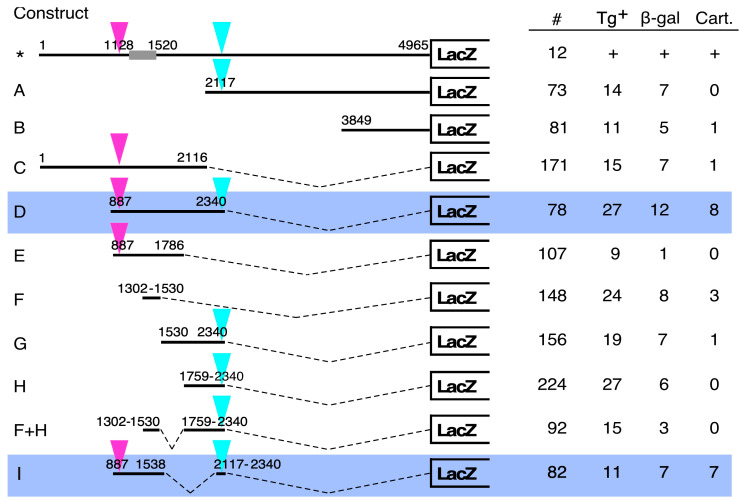
Deletion constructs and assays for reporter gene activity in transgenic mice. The first construct, labeled by an asterisk, depicts the structure of the reporter construct containing the 5 Kb *Hox8* upstream region that was previously published [11] but had not been analyzed at later developmental stages. For constructs produced in this study, all genomic DNA fragments from the *Hoxc8* locus were ligated to a *LacZ* reporter gene that is linked to the minimal promoter from the herpes simplex virus ICP4 gene [14]. This reporter gene is silent in the absence of a specific enhancer [14,15,19]. The addition of enhancer-containing DNA leads to reporter gene expression in transgenic mice, as analyzed at 15.5 days of development. The numbers of all embryos recovered from microinjections of a given construct are given, and the number of transgenic embryos (Tg+) was determined by a PCR assay for the *LacZ* transgene. β-galactosidase staining was detected macroscopically, and microscopic inspection of embryos and histological section was used to ascertain activity at specific sites. Construct F contains the previously identified so-called “early enhancer” [12], which produced staining in some skeletal elements that differed between three (out of eight transgenic) individuals and was deemed not active in the skeleton at the later stages we investigated. The minimal DNA sequences required for cartilage-specific enhancer activity consist of two elements (pink and turquoise triangles) located between nucleotides 887 and 1301 (pink) and 2117 and 2340 (turquoise). See the manuscript text for the rationale supporting this conclusion.

**Figure 3:**
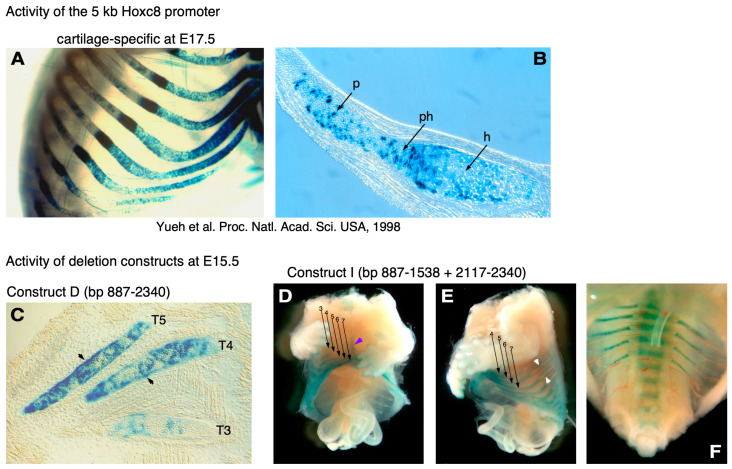
β-galactosidase activity in the cartilage of transgenic embryos. Transcriptional activation of *LacZ* reporter gene activity was assayed by staining for the reporter gene product β-galactosidase. (**A**) Transgenic embryo at E17.5 generated in the binary system [9] with tissue-specific β-galactosidase expression in the skeletogenic cells of the ribs, including the ventral rib cartilage. (**B**) The histological section of a rib reveals β-galactosidase staining in zones containing proliferative (p), prehypertrophic (ph), and hypertrophic chondrocytes (black arrows). (**C**) The section at an oblique angle from an E15.5 paraffin-embedded transgenic embryo carrying deletion construct D. β-galactosidase staining is apparent in the ribs (corresponding to proliferative zones of the ribs at T3, T4, and T5) and in perichondrial cells (arrows) but not in adjacent cells of mesodermal origin, such as, e.g., muscles. (**D**) A whole-mount partially eviscerated and β-galactosidase-stained transgenic embryo at E15.5 carrying construct I displays staining in the lower sternum (purple arrow) and the ribs (numbered in rostrocaudal direction by black arrows), with weaker intensity in segments T3 through T5 and a stronger signal in the lower ventral ribs. (**E**) Side view of another embryo transgenic for construct I; in addition to the black arrows for rostrocaudal numbering of ribs, white arrows point to areas of staining localized around the ossifying dorsal portions of ribs. (**F**) View of the lower body cavity of another construct I transgenic embryo from which the caudal region was removed; blue staining reveals reporter gene activity in the developing vertebrae at lower thoracic levels and in the corresponding rib heads of the lower floating ribs (visible in red are segmental blood vessels).

We then asked which specific regulatory elements control *Hoxc8* expression in cartilage. Our first objective was to ascertain cartilage specificity with a conventional transgene in which the *Hoxc8* upstream DNA is directly linked to the *LacZ* gene. Transgenic mice for such a construct had previously been generated [11] by Dr. C. Bieberich, who graciously provided them to us. We analyzed *LacZ* expression in embryos from several independent litters at 15.5 days of development. All animals that were transgenic for the construct (* in Figure 2) exhibited β-galactosidase activity. Reproducibly, staining was localized to the skeleton in a pattern congruent with the staining observed with the VP16-dependent binary transgenic system ([9], see also Figure 3A,B). These results confirmed with an independent transgene that the *Hoxc8* upstream region contains a genuine enhancer with specificity in chondrogenic cells of the developing skeleton. 

To map this enhancer within the 5 kb *Hoxc8* upstream region, we performed deletion analyses using the LacZ reporter linked to a generic viral minimal promoter [14,15], applying a stringent criterion for enhancer function: the activation of transcription in vivo from a heterologous promoter. Deletion constructs were produced by restriction digest or cloning (see Methods for details) and assayed in transient transgenic embryos (generated as the F0 generation). Embryos containing constructs A or B exhibited no specific activity of β-galactosidase in developing cartilage. A few animals exhibited β-galactosidase activity in multiple independent patterns, which lacked evidence of consistent enhancer activity. We then assayed the 5′ half of the *Hoxc8* upstream fragment (construct C), but again, found no specific activity in cartilage or skeletal structures. On the basis of these data, we considered the possibility that the restriction digest at nucleotide 2116 might have destroyed a critical site. Alternatively, elements on both the 5′ and 3′ halves (constructs A and C) may cooperate to form a cartilage-specific enhancer. 

To distinguish between these possibilities, we created construct D, which extends beyond the restriction cut site at 2116 bp. This reporter gene produced activity in cartilage in eight out of twelve transgene-expressing embryos (66.7%). The distribution of β-galactosidase staining was consistent between all eight embryos, indicating that the 1.4 kb insert of construct D contains genuine cartilage-specific enhancer activity. This was confirmed in histological sections, where β-galactosidase staining was found localized to skeletogenic cells (Figure 3H). No staining was observed in other somite-derived tissues, such as muscle. These results provide evidence that sequences between nucleotides 887 and 2340 are crucial for cartilage-specific enhancer function.

In an attempt to further reduce the DNA region containing cartilage active regulatory elements, we generated additional deletion constructs E, F, and G, which subdivided the 1.4 kb fragment of construct C. None of these smaller DNA constructs produced cartilage-specific β-galactosidase activity in transgenic embryos. Construct E exhibited staining in only one out of nine transgenic embryos. Construct F also did not confer consistent cartilage-specificity; the three transgenic embryos with some β-galactosidase activity in cartilage exhibited seemingly random patterns of activity in different skeletogenic structures, arguing against the presence of a strong enhancer between 1302 and 1530. A lack of activity of constructs G and H, encompassing basepairs 1530–2340 and 1759–2340, respectively, provided additional evidence against an active site around nucleotide 2116. From these results combined, we concluded that cooperation of (a) site(s) present between nucleotides 887 and 1786 and of (a) site(s) between nucleotides 1759 and 2340 are required for cartilage enhancer activity.

The latter fragment was then combined with the upstream fragment encompassing nucleotides 1302–1530. This fragment had previously been shown to contain transcription factor binding sites involved in “early” enhancer activity in transgenic embryos at E9.5, producing staining that was weaker than in the neural tube but still appreciable in the mesoderm [12]. Our rationale in combining this element with the 3′ part was that a mesoderm-specific element with activity in early embryos might cooperate with other DNA sequences to maintain gene expression in mesodermal derivatives at later stages of development. However, we found no cartilage-specific reporter gene expression from the combined construct (F + H). Therefore, the postulated interaction with an element located between 2117 and 2340 must involve sequences 5′ of bp 1302.

This proposition was tested with construct I, which combined sequences between nucleotides 887 and 1538 and 2117 and 2340. This construct also addressed the concern that a critical site could be located right at 1530 by extending the fragment 8 bps further downstream. We deleted region 1539–2116, which we concluded was not required on the basis of results from constructs F, G, H, and F + H. Construct I produced cartilage-specific activity in 5/7 (71%) of the transgenic embryos generated with this DNA fragment. Because the fragment comprising basepairs 2117–2340 is also contained in construct F + H, which is negative for enhancer activity, a functional interaction with the sequence between nucleotides 1302 and 1530 can be excluded. From the comparison of construct I to constructs G and H, the region spanning basepairs 1759–2116 can also be excluded as potentially relevant. Instead, the minimal sequences defined by our deletion analysis comprise the 416 basepairs of the 887–1302 upstream element and the 224 basepairs of the 2117–2340 downstream element. 

By assaying LacZ reporter activity by β-galactosidase staining, we observed a signal generated by construct I in the ribs (Figure 3E) and the developing vertebra (Figure 3F). It is interesting to note that in addition to the cartilaginous portions of the ribs, β-galactosidase staining was also found in the perichondrium of the ossifying dorsal parts of the ribs. This is consistent with the in situ hybridization results at E15.5 (see Figure 1H,H’) and indicates that enhancer activity is maintained in skeletogenic precursor cells, in addition to differentiating chondrocytes. A second intriguing feature of the cartilage enhancer is that even the minimal elements present in construct I exhibit region-specific activity in the developing skeleton (as did construct D), in that activity is prominent only below the level corresponding to thoracic vertebra 3 (see Figure 3D,E). Thus, in all constructs with enhancer activity, the regulatory element also conferred regional restriction along the anterior–posterior axis.

**Figure 4:**
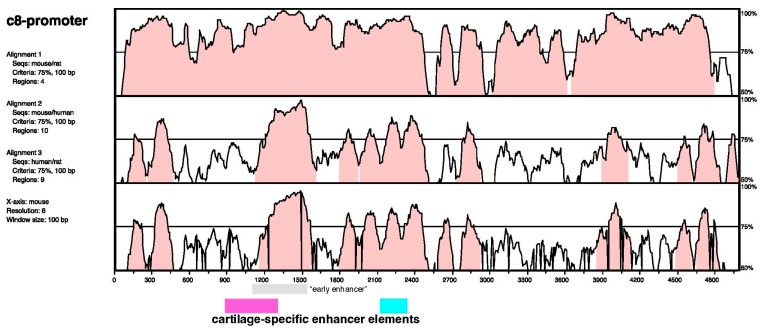
Evolutionary comparison of the *Hoxc8* upstream flanking region, including the developmentally early enhancer and the chondrocyte-specific enhancer. VISTA plot for pairwise comparisons of rat–mouse (top row), mouse–human (middle row), and rat–human (lower row) *Hoxc8* upstream sequences. Several regions of conservation (a window size of 100 bp) across species above 75% (red shading) are evident, one of which contains the previously described *Hoxc8* “early enhancer” (gray bar below the comparison plot). Pink- and turquoise-colored bars below the comparison plot depict the location of the cartilage-specific enhancer elements defined in our studies, as depicted in Figure 2.

**Figure 5:**
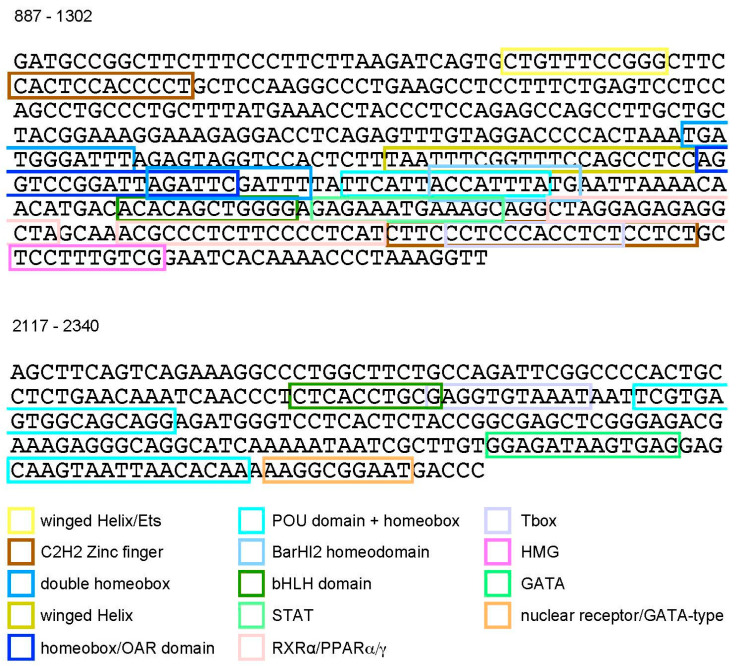
Sequence analysis of the *Hoxc8* cartilage-specific enhancer and transcription factor binding sites. Sequences of the minimal elements defined from the results depicted in Figure 2. Motifs for potential transcription factor binding were collected, identified by both JASPAR^2018^ and JASPAR^2020^ versions, and visualized in the UCSC genome browser.

To gain insight into the transcriptional mechanisms involved in the regulation of the cartilage-specific enhancer, we performed sequence analyses of the *Hoxc8* upstream region. Cross-species comparisons (Figure 4) revealed evolutionary conservation of non-coding sequences with particularly high similarities in the areas between bps 1100 and 1650 and 1800 and 2500. The previously described “early enhancer” ([12], the gray box in Figure 4) is known to be highly conserved among vertebrates [20]. Our analysis reveals additional areas of sequence conservation, which cover the interacting elements of our cartilage-specific enhancer (pink and turquoise bars in Figure 4). Analysis of the mouse sequence, annotated by the Jaspar transcription factor binding site collection, predicts the presence of potential binding sites for transcription factors of diverse classes (Figure 5): both elements of the cartilage-specific enhancer contain binding motifs for POU-domain/homeodomain transcription factors, Tbox transcription factors, and bHLH transcription factors. Unique to the upstream element are motifs for winged helix-domain transcription factors, double homeoboxes, BarH-like homeoboxes, and homeobox/OAR-domain transcription factors, a motif for nuclear receptor binding (NR2C2), and most notably, an element for binding of high-mobility group transcription factors, which are known to be required for initiation and maintenance of cartilage-specific gene transcription. Unique to the downstream element are motifs for the binding of GATA factors and nuclear receptors of the GATA class. Several of the motifs overlap, precluding unequivocal attribution of particular transcription factor binding activities or combinations thereof.

## 4. Discussion

This study aimed to identify the regulatory element(s) in the *Hoxc8* gene that are necessary and sufficient for controlling gene transcription in skeletogenic cells at later stages of embryonic development. Using a transient transgenic reporter approach in mice, we show that cartilage-specific enhancer activity requires the cooperation of two sequence elements in the 5 Kb upstream region of the *Hoxc8* gene: a 416 basepair element (encompassing bps 887–1302) and a 224 basepair element (comprising bps 2117–2340). The localization of these elements relative to each other does not seem to affect their enhancer activity, as they are 579 bp farther apart from each other in the native configuration and in construct D compared to construct I. The latter two constructs also indicate that the cartilage-specific enhancer maintains activity, even at variable distances to the transcription start site, at least with the heterologous promoter tested. Thus, by employing the transient transgenic in vivo assay, we have identified a bona fide chondrocyte-specific enhancer upstream of the *Hoxc8* gene.

The cartilage-specific activity of the enhancer is observed in the actively growing ventral sections of the developing ribs at E15.5, with reporter staining maintained in perichondrial cells in the rib sections that have started hypertrophy and ossification (see the open arrows in Figure 3A,D). Activity is also evident in the developing sternum and the vertebrae (Figure 3D,F), indicating a role for the enhancer in all skeletal elements at a given segmental level. Of note, the more intense reporter staining in the binary transgenic system (panels A, B) is likely due to the amplification of transcriptional activation by the VP16 transactivator [9,19]. Within cartilaginous elements, not all cells are uniformly stained to the same extent (see Figure 3B,C), indicating tissue-specific “mosaicism”, which was also observed when the *LacZ* reporter gene was knocked into the endogenous *Hoxc8* locus [6]. Thus, the activity of our enhancer elements recapitulates cellular patterns of expression activity that are also observed in the native *Hoxc8* locus. However, which DNA sequences in particular control the activity remains to be tested by targeted mutagenesis.

Combinatorial action of multiple elements in transcriptional regulation has been described for developmental enhancers in multiple *Hox* genes, such as, for example, *Hoxa7* [21], *Hoxb4* [22] *Hoxb7* [23], *Hoxb8* [24], *Hoxd11* [25], and in the *Hoxc8* gene itself. Shashikant et al. [12] defined a 335 bp enhancer (nucleotides 1128–1462) with activity in the neural tube that initially was described as encompassing four interacting elements and was later extended by 64 bp to include a fifth element that confers activity early in mesodermal cells (1128–1526; [26]). Binding motifs were identified for Cdx transcription factors, forkhead transcription factor Foxa2/HNF3, Hox transcription factors, and HMG domain-containing proteins. These sites are closely spaced within a promoter–proximal 200 bp fragment (nucleotides 1328–1527), the so-called “early enhancer” [12], with activity in developing neural tube posterior to the 10th somite pair and in mesoderm posterior to the 14th some pair. The deletion of this 200 bp sequence from the endogenous mouse *Hoxc8* locus in vivo [27] revealed that it is not required for the transcription of the endogenous *Hoxc8* gene or regional specificity, but the “early enhancer” sequence plays a role for proper temporal regulation.

Indeed, the cartilage-specific activity of our enhancer does not require these “early enhancer” sequences, as evidenced by a lack of activity in the developing skeleton with construct F, which comprises the “early enhancer”. The addition of this DNA segment to the 224 bp downstream element we defined (in construct F + H) also failed to stimulate reporter transcription in chondrogenic cells. Thus, we have delineated a tissue-specific enhancer with activity at later stages of development that is distinct from early enhancers, comprising elements spaced both more distal from and more proximal to the transcription start site. Our sequence analysis reveals potential binding sites for many types of transcription factor molecules, among them HMG domain proteins, and Pbx/Hox transcription factor complexes. The HMG domain proteins Sox9, Sox5, and Sox6 are well-known transcriptional regulators in chondrocytes [28]. The implication of particular transcription factors in the activation and maintenance of activity of the *Hoxc8* cartilage-specific enhancer will have to await further investigation by mutational analysis.

Intriguingly, even though the enhancer displayed strong specificity for cartilage, it maintained a regional restriction to the thoracic and lumbar/sacral regions of the developing skeleton, with weaker staining in ribs in the T3 through T5 segments and more prominent staining in T6 and T7 ribs, continuing more posteriorly. This is consistent with increasingly stronger staining in the lower sternal ribs observed in mice where the LacZ gene is inserted into the *Hoxc8* locus [6]. These and other authors already noted that the rostral boundary of reporter gene expression at later developmental stages (E12.5) was located between T3 and T4 skeletal elements [5,6] and at the newborn stage extended to T1, including the first pair of ribs [6]. This is consistent with our findings for *Hoxc8* upstream region activity with the binary transgenic system in newborn mice [9]. The mechanisms underlying this anterior shift compared to earlier developmental stages are unknown but could involve a lack of activity of the “early enhancer” at later stages, either due to the absence of relevant transcription factors in chondrogenic cells or epigenetic modifications that make it inaccessible. Alternatively, the earlier posterior restriction in skeletogenic precursor structures (i.e., the somites) could be mediated by a silencing element, preventing the action of the “early enhancer” in anterior regions. If such a repressive element were to exist, it is obviously no longer active by the time chondrogenic cells differentiate in the thoracic and lumbosacral regions of the developing skeleton.

It is noteworthy that the cartilage-specific *Hoxc8* enhancer that we have identified is also active in perichondrial cells (see the arrows in Figure 3C,E) before ossification is completed. The perichondrium is known to play a critical role in catch-up growth [29] and the regeneration of long-bone cartilage, such as rib cartilage [30]. Our enhancer will now allow studies that follow *Hoxc8* expression in these cells during the regeneration process. It is also intriguing that despite widely documented functional roles of *Hox* genes in adult tissues and cancer [31,32,33,34,35,36,37,38,39,40,41,42,43,44], few attempts have been reported to identify regulatory elements controlling *Hox* gene expression in specific adult cell types in vivo. For example, Tabaries et al. [45] identified regulatory sequences in the *Hoxa5* gene with strong activity in thoracic prevertebrae, but whether the activity is maintained through late development or postnatally has not been investigated. Similarly, Pruett et al. [34] described regionally distinct reporter activity specifically in vascular smooth muscle cells driven by sequences from the *Hoxa3* and *Hoxc11* genes, but the responsible regulatory elements have not been identified. Thus, our study is one of only a few reports that characterize regulatory enhancer elements for cell-type-specific *Hox* gene expression through an in vivo approach. This now makes it possible to investigate the activity and regulation of this enhancer and, by implication, its transcription regulatory mechanisms, in postnatal skeletogenic processes, such as chondrocyte proliferation and differentiation during the growth of the skeleton, hormonal regulation of bone growth completion during puberty, cartilage and bone regeneration after injury and bone fracture repair, and transplantation and bioengineering approaches to cartilage tissue regeneration.

## Data Availability

The datasets used and analyzed during the current study are available from the corresponding author upon reasonable request, in compliance with institutional policies of Pennington Biomedical Research Center/Louisiana State University System. DNA constructs will be made available upon request.
